# Supplementary material for: Identification of photoperiod-induced specific miRNAs in the adrenal glands of Sunite sheep (Ovis aries)
Source: Front Vet Sci. 2022 Jul 22;9:888207. doi: 10.3389/fvets.2022.888207 (PMC9354845; doi:10.3389/fvets.2022.888207)
Supplement: Supplementary file 1 [file Data_Sheet_1.ZIP › Supplementary file/miRDeep2 file.zip!/miRDeep2 file/Know_Structure/SPLP42A1/oar-mir-329a.pdf]

miRBase precursor : oar-mir-329a
Total read count : 84
oar-miR-329a-5p read count : 7
oar-miR-329a-3p read count : 76
remaining reads : 1

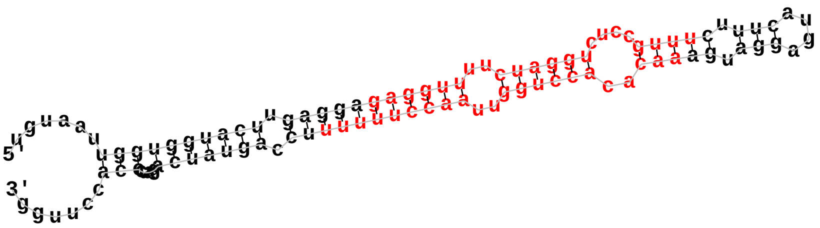

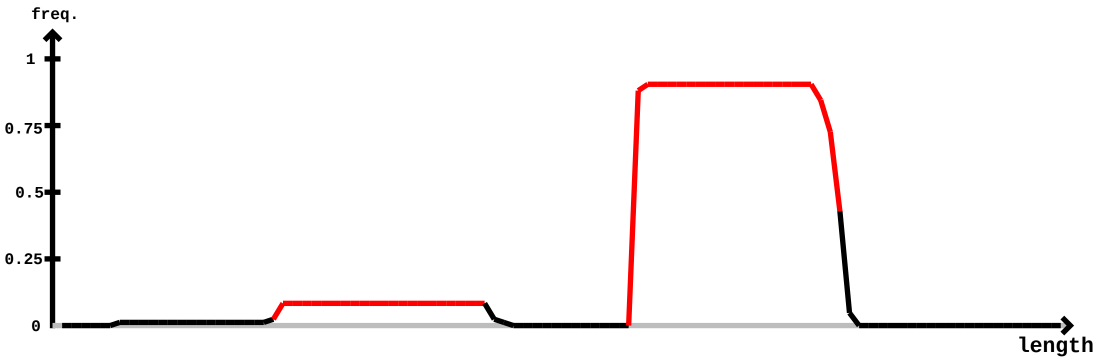


```
                                   oar-miR-329a-5p
                                                                        oar-miR-329a-3p
5'- uguaauuggugguacuugaggagagguuuucuaggucuccguuucuuucaugaggaugaaacacaccugguuaaccuuuuuuccaguaucagaucccaccuugg   -3'   exp
    .......(((((((((.(((((((((((..((((((....(((((.(((....))).)))))..))))))..))))))))))).)))).......)))))....   reads   mm   sample
    .....uuggugguacuugagga..................................................................................   1       0    seq
    .....................agagguuuucuaggucuccguuu............................................................   1       0    seq
    ......................gagguuuucuaggucuccguuu............................................................   4       0    seq
    ......................gagguuuucuaggucuccguuuc...........................................................   1       0    seq
    ......................gagguuuucuaggucuccguuucu..........................................................   1       0    seq
    ...........................................................aacacaccugguuaaccuu..........................   5       0    seq
    ...........................................................aacacaccugguuaaccuuu.........................   10      0    seq
    ...........................................................aacacaccugguuaaccuuuu........................   25      0    seq
    ...........................................................aacacaccugguuaaccuuuuu.......................   31      0    seq
    ...........................................................aacacaccugguuaaccuuuuuu.....................   3       0    seq
    ............................................................acacaccugguuaaccuuuu........................   1       0    seq
    ............................................................acacaccugguuaaccuuuuuu......................   1       0    seq
```
